# A Strip Adjustment Method of UAV-Borne LiDAR Point Cloud Based on DEM Features for Mountainous Area †

**DOI:** 10.3390/s21082782

**Published:** 2021-04-15

**Authors:** Zequan Chen, Jianping Li, Bisheng Yang

**Affiliations:** 1 State Key Laboratory of Information Engineering in Surveying, Mapping and Remote Sensing, Wuhan University, Wuhan 430079, China; zeeqchen@whu.edu.cn (Z.C.); lijianping@whu.edu.cn (J.L.); 2 Engineering Research Center for Spatio-Temporal Data Smart Acquisition and Application, Ministry of Education, Wuhan University, Wuhan 430079, China

**Keywords:** strip adjustment, low-cost UAV, DEM, LiDAR point cloud, point-to-plane ICP, registration, mountainous areas, terrain features

## Abstract

Due to the trajectory error of the low-precision position and orientation system (POS) used in unmanned aerial laser scanning (ULS), discrepancies usually exist between adjacent LiDAR (Light Detection and Ranging) strips. Strip adjustment is an effective way to eliminate these discrepancies. However, it is difficult to apply existing strip adjustment methods in mountainous areas with few artificial objects. Thus, digital elevation model-iterative closest point (DEM-ICP), a pair-wise registration method that takes topography features into account, is proposed in this paper. First, DEM-ICP filters the point clouds to remove the non-ground points. Second, the ground points are interpolated to generate continuous DEMs. Finally, a point-to-plane ICP algorithm is performed to register the adjacent DEMs with the overlapping area. A graph-based optimization is utilized following DEM-ICP to estimate the correction parameters and achieve global consistency between all strips. Experiments were carried out using eight strips collected by ULS in mountainous areas to evaluate the proposed method. The average root-mean-square error (RMSE) of all data was less than 0.4 m after the proposed strip adjustment, which was only 0.015 m higher than the result of manual registration (ground truth). In addition, the plane fitting accuracy of lateral point clouds was improved 4.2-fold, from 1.565 to 0.375 m, demonstrating the robustness and accuracy of the proposed method.

## 1. Introduction

The unmanned aerial vehicle (UAV) Light Detection and Ranging (LiDAR) system is a multi-functional and highly automatic system to obtain terrain information. Compared with traditional remote sensing technology, ULS (unmanned aerial laser scanning) has the advantage of active measurement. As a new revolutionary measurement technology, it has attracted wide interest from industry and academia [1]. This technology has been widely used in 3D vegetation mapping [2], disaster management [3,4], forestry inventory [5,6,7,8], power line inspection [9,10], 3D city development [11], etc.

ULS [12] is a complex multi-sensor integrated system composed of multiple components such as Global Navigation Satellite System (GNSS), Inertial Navigation System (INS), and laser scanners. Systematic errors caused by the integration of these components affects the accuracy of the collected data. In practice, an area of interest usually needs to be covered by several overlapped parallel LiDAR strips collected by ULS. However, due to the systematic error and the attitude error of the position and orientation system (POS) [13], offset of the same object exists in adjacent LiDAR strips. These offsets not only have negative impacts on the point cloud accuracy, but also in application. For example, if unadjusted strips are used to directly generate a digital elevation model (DEM), the terrain surface will be discontinuous and incomplete, which cannot be further utilized. Therefore, eliminating the discrepancy between different strips to achieve global consistency is indispensable [14].

Strip adjustment is an effective way to eliminate these discrepancies. Existing strip adjustment methods for eliminating the discrepancy of laser scanning data generally fall into two categories: system-driven strip adjustment and data-driven strip adjustment [15,16]. System-driven strip adjustment is mainly based on estimating the calibration parameters to compensate for the sensor placement errors. However, due to the confidentiality of the LiDAR system, it is difficult for users to obtain the raw observations (such as angle and distance) directly. Usually, users can only obtain the 3D point clouds, which leads to difficulties in the use of the system-driven strip adjustment method. On the contrary, the data-driven strip adjustment method does not need to access the raw LiDAR data and GNSS/inertial measurement unit (IMU) data. It utilizes the corresponding features in the adjacent strips and achieves accurate registration between strips using rigid transformations. This kind of method also has the defect that it is not rigorous in theory because it does not consider the sensor model of LiDAR.

In recent decades, scholars have conducted extensive and in-depth research on strip adjustment, which can eliminate the discrepancy between adjacent strips. Although the reported methods have been successfully applied to eliminate systematic errors between the ULS strips in urban scenes, they still have difficulties in dealing with laser scanning data from mountainous areas because of the complex terrain, large vegetation coverage, few artificial buildings, and lack of reliable homonymous features. Thus, a data-driven strip adjustment method based on topographic features for mountainous areas is proposed in this paper, which can be applied to the strips collected by ULS in mountainous areas.

The main contributions of this paper are two-fold:

1. We propose DEM-iterative closest point (ICP), a variation of point-to-plane ICP [17], taking the mountainous characteristics into account. Compared with the classic point-to-plane ICP, DEM-ICP has the advantages of good robustness, high registration accuracy, and fast convergence speed.
2. We utilize graph-based optimization following DEM-ICP, achieving accurate strip adjustment in mountainous areas. This is an effective means of addressing the disadvantages of existing methods, which are difficult to use in mountainous areas that lack the corresponding features. The proposed data-driven strip adjustment method only requires 3D point clouds, so it is suitable for a wide range of applications.

The rest of the paper is organized as follows: Section 2 reviews the literature on strip adjustment. In Section 3, the workflow of the proposed method is demonstrated. Then, the comprehensive experiments are described in Section 4. Finally, the conclusions and future research directions are presented in Section 5.

## 2. Related Work

Due to the existence of systematic error, the data collected by LiDAR systems cannot be directly applied to production. Many strip adjustment methods have been proposed to eliminate the systematic error of LiDAR systems. Chan et al. [18] proposed a system calibration method based on planar features and catenary features, which could simultaneously estimate multiple boresight angles of different laser scanners. In their method, least-squares adjustment (LSA) was used to estimate the parameters of a land-based mobile mapping system (MMS). Experiments showed that the introduced three-dimensional catenary curve model could improve the overall accuracy when there were long- hanging cables. In [19], point and plane features were extracted and matched with the ground control features generated from accurately georeferenced terrestrial laser scanners (TLS) data. Based on LSA, their method could simultaneously estimate the boresight and lever-arm parameters of the sensors mounted on an MMS. However, because the perspective and density of the MMS and UAV LiDAR system are different, the existing strip adjustment methods for MMS cannot be directly applied for UAVs.

Regarding airborne laser scanning (ALS), strip adjustment can be simplified as the one-dimensional correction problem in a flat area, only considering the error of the elevation. Crombaghs et al. [20] designed a practical one-dimensional strip adjustment model, which only checked the data elevation, and was successfully applied to the production of DEM data. However, Vosselman and Maas [21] believed that the error of airborne LiDAR data in the horizontal direction was greater than that in the vertical direction, and elevation adjustment could not completely eliminate the system error. Therefore, the method of only focusing on elevation without considering the difference of horizontal coordinates cannot meet the production and application needs of strip adjustment in most situations. Subsequently, most of the methods proposed by scholars aim to correct the three-dimensional errors. The existing strip adjustment methods can be generally categorized into system-driven and data-driven methods.

### 2.1. System-Driven Strip Adjustment Methods

The system-driven strip adjustment method estimates the error parameters based on the system error source. Skaloud and Lichti [22] proposed a rigorous method for estimating the calibration parameters (the three bore-sight angles and the range-finder offset) in ALS (airborne laser scanning), which can work with voluminous ALS and INS/GPS (Global Positioning System) data. This method was robust and accurate when there were sufficiently strong geometries. Junior and Santos [23] estimated the calibration parameters by restricting the centroid of the segmentation plane to the corresponding segmented plane, and then used the proposed corresponding point-to-plane strategy to refine the boresight angles. Experiments showed that this approach is slightly better than Skaloud and Lichti’s method [22] when the geometric constraint was also included. Habib et al. [24] presented a “simplified method” and a “quasi-rigorous method”, which do not rely on the empirical and proprietary procedures. The proposed methods were relatively easy to be implemented and could be applied to a variety of terrain coverage. Ravi et al. [25] used self-made high reflection calibration boards to increase the conjugate feature extraction efficiency. Furthermore, they proposed a procedure to calibrate the mounting parameters of the multiunit LiDAR system. The experiments indicated that by adopting the optimal configuration, the calibration result was better than the expected accuracy. Zhang et al. [26] considered the influence of the low-precision POS system error and proposed an aerotriangulation-aided LiDAR strip adjustment (AT-aided LSA) method, which required auxiliary information such as aerial images in the same area.

The self-calibration method is more feasible because it does not demand extra referencing data during calibration. Zhang et al. [27] proposed a virtual tie point model to solve the problem of points corresponding in the discrete point clouds. Employing the intensity data, this self-calibrated method could extract corresponding points automatically and was proved to be feasible and effective by experiments. Li et al. [16] developed an automatic boresight self-calibration method, in which the boresight angles were expressed in the direct geo-referencing equation. The ICP [28] algorithm was used to search the point-to-point correspondences between strips. Their method could significantly reduce the root-mean-square errors (RMSEs) of misalignments of the mobile LiDAR scanning (MLS) systems and ULS systems. In [22], a self-calibration method was also reported.

### 2.2. Data-Driven Strip Adjustment Methods

Many scientific studies on the data-driven strip adjustment method have also been reported. Wang [29] used “approximate control points” and “approximate connection points” to establish a regional network adjustment model, which was suitable for airborne LiDAR strip adjustment in a variety of different surface conditions. Yang et al. [30] proposed a multi-view registration method based on semantic feature points, which was marker-free and achieved good registration performance. In [31], an airborne LiDAR strip adjustment method based on planar features extracted from buildings was introduced. They applied the minimum Hausdorff distance (MHD) to match the buildings and planar features. Experiments indicated that the proposed method was more automatic than that of Wu and Fan [32]. You and Lee [33] introduced surface feature strength data derived from the tensor voting method into strip adjustment. Based on the partial least squares method, it improved the accuracy significantly when the surface feature strength data and height data were utilized together.

Usually, the amount of data collected by airborne LiDAR is very large. If the proposed algorithm lacks automation, its application will be labor-intensive. In Lee et al. [34], an automatic method utilizing changes in local height variations was reported, which used the contour tree (CT) to represent local height changes to find a suitable initial transformation and then optimized the function parameters using the ICP algorithm. This method did not acquire any preprocessing, and could eliminate the discrepancies significantly. Rentsch and Krzystek [35] utilized a five-parameter adjustment model to eliminate LiDAR system errors, relying on the match of the roof plane. Glira et al. [36] proposed an automatic strip adjustment method, which iteratively and directly established correspondences between points of overlapping ALS strips. The experiments showed that this method could individually correct the trajectory errors of strips and calibrate the entire ALS multisensor system in real time. By considering the time-dependent trajectory errors and modeling them by natural cubic splines, they extended their previous work [36] to achieve a more accurate georeference of point clouds [37].

In addition, methods were also designed for the adjustment of underwater LiDAR strips. In Ji et al. [38], a coarse-to-fine strip adjustment method was developed for airborne LiDAR bathymetry (ALB). Due to the monotonous underwater environment targets, the point cloud density collected by ALB is low, which makes it difficult to mosaic adjacent strips. Therefore, they proposed an improved alpha shapes algorithm to rapidly and accurately detect the overlapping area between strips. Based on the non-rigid ICP algorithm and least-squares trend surface fitting algorithm, the proposed method achieved good performance.

Overall, because the raw observation data is usually difficult to obtain by end-users, the data-driven strip adjustment method is user-friendly compared with the system-driven method. In addition, most of the existing methods rely on the homonymous features between adjacent strips, and may not be able to eliminate the systematic errors among the strips in mountainous areas lacking distinctive features. Thus, this paper proposes a data-driven strip adjustment method for ULS strips collected in mountainous areas.

## 3. Methodology

**Figure 1:**
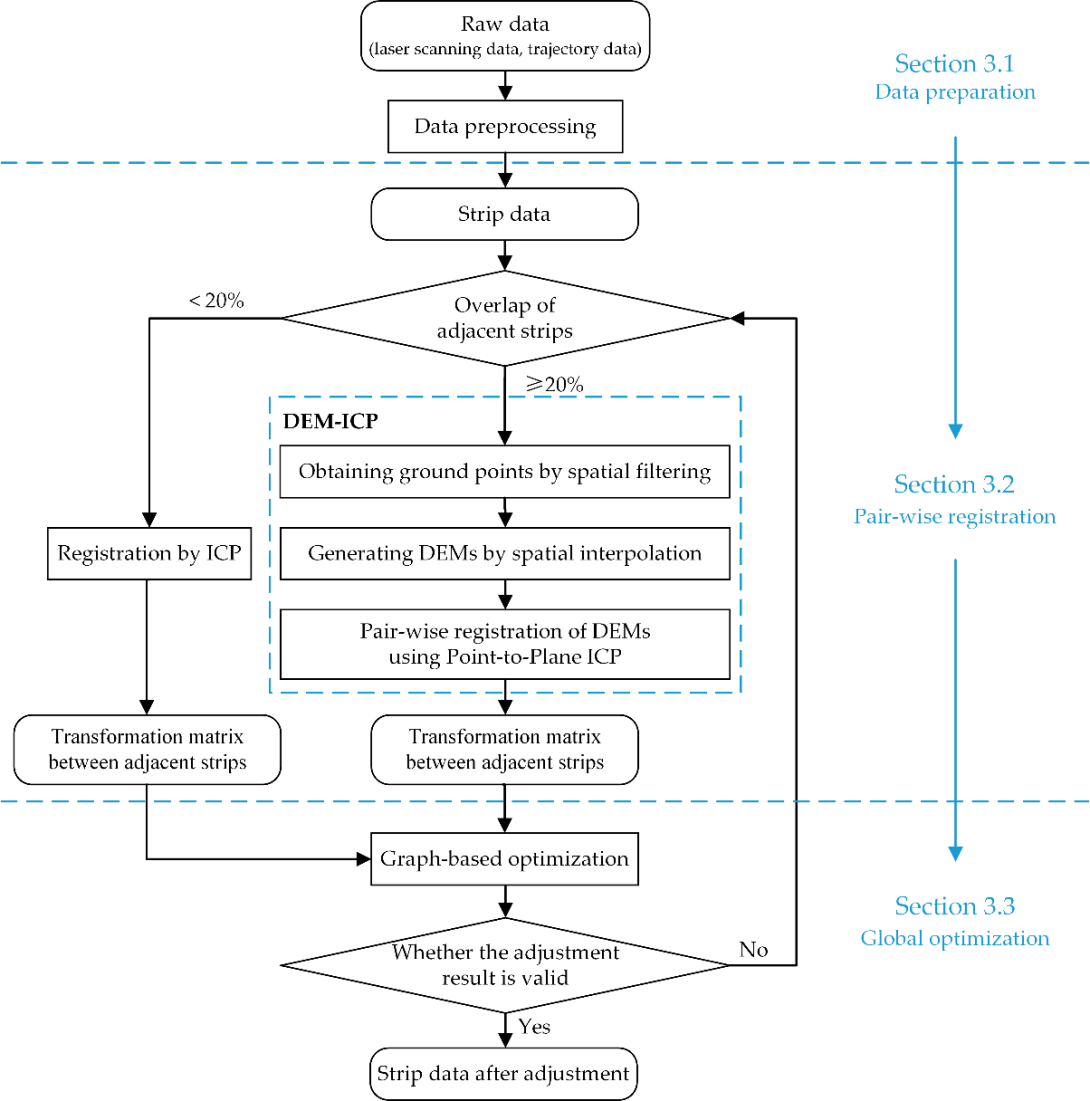
The workflow of the proposed method.

The proposed method mainly includes three steps: data preprocessing, DEM-ICP registration, and graph-based optimization. The main process, DEM-ICP, can be further divided into spatial filtering, ground point interpolation, and pair-wise registration of DEM. The complete workflow is shown in Figure 1. Some of the symbols used in this section and their brief descriptions are shown in Table 1.

### 3.1. Data Preprocessing

Because of the load and budget constraints of low-cost UAV, the POS accuracy is relatively low [39]. POS is the key system of UAV trajectory navigation and UAV pose recording. During data acquisition, the cumulative effect of the systematic error is more obvious, and the accuracy of the collected point cloud is lower.

NRLI-UAV [40] is a two-step non-rigid registration method and was introduced to preprocess the original point clouds. Firstly, the GNSS and IMU-aided structure from motion (SfM) was used to obtain accurate image orientation and correct the errors of the low-precision POS. Secondly, by setting the oriented images as the reference, the discrepancy between the depth maps derived from SfM and the raw laser scans was iteratively minimized to achieve accurate registration between the images and the LiDAR point clouds.

### 3.2. Pair-Wise Registration

The DEM-ICP method proposed in this paper mainly includes the following three steps: spatial filtering, ground point interpolation, and pair-wise registration of DEM.

#### 3.2.1. Spatial Filtering

Terrain features are a unique sign of the undulating ground surface. To obtain the characteristics of the terrain, it is necessary to filter out the non-ground points and reserve the ground points.

In recent decades, many studies on airborne LiDAR point cloud filtering have been carried out, which can be divided into two types: those based on point entities and those based on segment entities [41]. However, the method only using one entity cannot easily achieve a good filter result in various complex scenes [42]. Fortunately, the combination of different entities (e.g., points and segments) can achieve better filtering results.

In this study, the two-step adaptive extraction method proposed by Yang et al. [43] was used to acquire the ground points, which first classifies the points into a set of segments and one set of individual points, and then extracts the breaklines from the ground points to generate a high-quality DEM. This method has good filtering performance and can filter out non-ground points while retaining ground points to the maximum extent, which is convenient for subsequent interpolation of ground points.

#### 3.2.2. Ground Point Interpolation

Due to the filtering out of the non-ground points, many holes exist in the filtered ground data. The continuous and complete DEM data should be used for registration, so it is necessary to interpolate the ground points. In this study, the DEM data was organized by raster. The quality of the interpolation algorithm will affect the accuracy of registration. Commonly used high-quality interpolation methods are kriging [44], natural neighbor interpolation (NNI) algorithm [45], etc.

After comparing different interpolation algorithms, NNI was adopted to interpolate the ground points due to its best performance in the experiments. The NNI algorithm is a local interpolation method based on the Voronoi diagram, in which the value of an interpolation point depends on the value of its natural neighbor points. Let the interpolation point *x* have *n* natural neighbor points, which are *p*_1,_ *p*_2*,…,*_ *p_n_*. Then the value of *f*(*x*) can be expressed as:

(1) $$f\left(x\right)=\overset{n}{\underset{i=1}{\sum }}w_{i}f(p_{i})$$

where $w_{i}$ is the weight of the corresponding point $p_{i}$, $f(p_{i})$ denotes the value of the natural neighbor point $p_{i}$.

**Figure 2:**
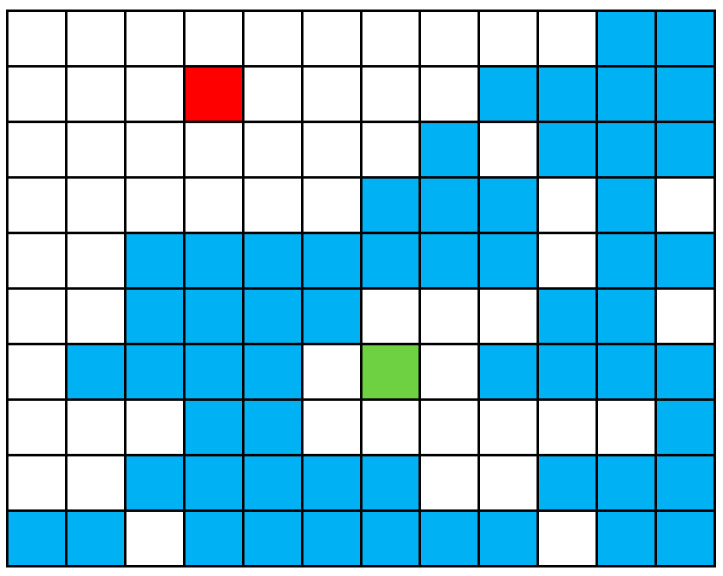
Illustration of the DEM interpolation. Blue points are ground points, green and red points are points to be interpolated. Here, raw data exist around the green point, so a more accurate prediction can be obtained during interpolation. On the contrary, the red point can only rely on the data in the lower right corner to make predictions. There is no original data in the upper left corner, so it is difficult to obtain correct predictions.

It should be noted that during the interpolation, because the value of the points to be interpolated in the edge of DEM can only be predicted by the data in one direction, the interpolation result is often incorrect (as shown in Figure 2). The existence of these wrong edge points will affect the registration of DEM. It is a simple but effective method to remove a certain width of the edge from the interpolated DEM data. Doing so will not reduce the range of the experimental data, because the range of DEM data after interpolation will be larger than that before interpolation. In other words, only the extra part due to interpolation is cropped. The specific cutting width needs to be determined according to the situation of the DEM.

#### 3.2.3. Pair-Wise Registration of DEM

Compared with the disordered massive point cloud, DEM data has the characteristics of small data quantities and ordered arrangement, which can effectively express the topographic features. Therefore, using DEM instead of the raw points for registration of point cloud in mountainous areas can improve efficiency. To acquire a more robust registration result [46], the point-to-plane ICP algorithm was adopted to register DEM data, which first estimates the normal vector of the target point cloud, and then replaces the point-to-point distance in the ICP algorithm with the point-to-plane distance, resulting in better robustness and faster convergence. The point-to-plane ICP is a commonly employed strategy, whose error function E(R,t) is calculated as Equation (2).

(2) $$E\left(R,t\right)=\frac{1}{n}\overset{n}{\underset{i=1}{\sum }}{\left\|\eta_{i}[q_{i}-(Rp_{i}+t)]\right\|}^{2}$$

where *n* is the number of the nearest neighbor point pairs, $q_{i}$ is a point in the source cloud *Q*, $p_{i}$ is a point in the target cloud *P*, the corresponding nearest point of $q_{i}$. $\eta_{i}$ is the normal vector of the tangent plane of a point $p_{i}$. *t* denotes the translation vector, which can be expressed as ${\left[t_{x},t_{y},t_{z}\right]}^{T}$. *R* is a 3 × 3 rotation matrix.

For the point clouds without scaling, the registration between adjacent strips only needs to consider the rotation and translation in three-dimensional space. The rotation and translation transformation between adjacent LiDAR strips can be directly represented by a 4 × 4 transformation matrix *T*:

(3) $$T=\left[\begin{array}{cc} R & t\\ 0^{T} & 1 \end{array}\right]$$

It has been found that the overlap between strips is an important factor affecting the registration results. Hence, the selection of registration methods based on the overlap is important for good results. The DEM-ICP method proposed in this paper can obtain better registration results when the overlapping area has distinctive terrain features and the overlap is more than or equal to 20% (this threshold derives from the overlap test results in Section 4.2.1). To this end, different registration strategies are chosen according to the overlap of adjacent strips. When the overlap of adjacent strips is more than 20%, the DEM-ICP method is preferred; when the overlap is relatively small, but there is a good initial alignment between adjacent strips, they are directly registered by ICP algorithm.

The maximum search distance (MSD) is a key parameter for registration. Regarding two points located in the source and the target point clouds, if the distance between them is greater than the MSD, they will not be used as a corresponding point pair. If the initial registration parameters are inaccurate, the MSD should be set larger to prevent local optimization.

There are two stop criteria of the registration, as follows:

- Let $R_{n}$ and $t_{n}$ be the rotation matrix and translation vector obtained by the $n^{th}$ iteration. Then, if $\|t_{n}-t_{n-1}\|<t_{0}$ and $tr\left(R_{n-1}^{T}\cdot R_{n}\right)<r_{0}$ *tr*(·) is the trace of a matrix), the iteration will be stopped. Here, $t_{0}$ and $r_{0}$ are two thresholds, whose empirical values are 0.05 m and 1 × 10^−3^°, respectively.
- Let K be the maximum number of iterations. When the ICP algorithm iterates K times, the registration is stopped.

### 3.3. Graph-Based Optimization

**Figure 3:**
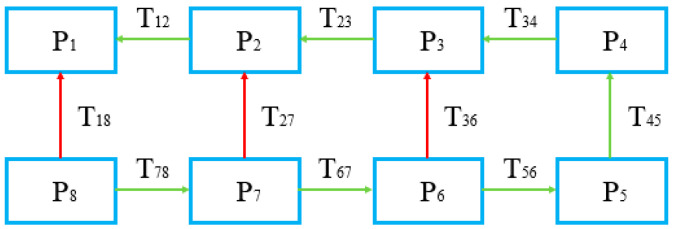
Illustration of graph-based optimization. The blue rectangles represent the strip data, and the arrows represent the registration relationship between them. Among these, the green arrows are the necessary observations (not unique) and the red arrows are the redundant observations.

During registration, each pair of adjacent LiDAR strips can be effectively registered once to obtain a transformation matrix *T* (as shown in Figure 3). In the problem of strip adjustment without control points, only *n* − 1 necessary registrations (green arrow in Figure 3) are needed to register *n* strips together. When the registration relationship between strips can form one or more loops, there will be redundant registration relationships (red arrow in Figure 3). Therefore, we can use the graph-based optimization method to adjust the registration results to gain more reasonable results.

Graph-based optimization solves global adjustment problems using a graph [47]. A graph consists of several vertices and edges. The vertices represent optimization variables and the edges represent error terms. After gaining the transformation matrix *T* between adjacent strips by registration, a graph can be constructed by taking the pose of each point cloud as the vertex and the registration relationship between them as the edge, and then the solution can be solved by graph-based optimization.

In the problem of strip adjustment without control points, the pose of a strip can be set as a unit matrix *I*_4×4_, then the pose of other strips can be obtained by the registration relation *T* between adjacent strips:

(4) $$P_{n}=T_{\left(n-1\right)n}\cdot P_{n-1}$$

where $P_{n}$ denotes the pose of the *n*^th^ strip, a 4 × 4 matrix of the same form as ***T*** shown in Equation (3). $T_{\left(n-1\right)n}$ represents the transformation matrix gained by registering the *n*^th^ strip to the (n − 1)^th^ strip.

The aim of the optimization is to minimize the following equation:

(5) $$minF\left(x\right)=\overset{n}{\underset{k=1}{\sum }}e_{k}{\left(x_{k}\right)}^{T}\Omega_{k}e_{k}(x_{k})$$

where F(x) is the function to be optimized, $e_{k}{(x}_{k})$ denotes the error of the *k*^th^ edge *x_k_*, and $\Omega_{k}$ is the information matrix, which is the inverse of the covariance matrix. After constructing the graph, the nonlinear least-squares problem can be solved by gradient descent algorithms such as the Levenberg–Marquardt method [48,49].

Graph-based optimization is a global method to minimize the discrepancies between all strips. If there is a big difference between the result of a strip after graph-based optimization and that before optimization, it means that there are outliers in the pairwise registration. Let $m_{ij}$ be the MSD of strip *i* and strip *j* during registration, the point cloud after registration as the source point cloud, and the point cloud after graph-based optimization as the target point cloud. Then, each point $p_{k}$ in the target point cloud searches for its nearest point in the source point cloud, and calculates the distance $d_{k}$ between them. If $d_{k}<m_{ij}$, this pair of points is marked as a valid point pair. Then *D = D + d_k_*, *n = n* + 1, where *D* is the sum of the distance of the valid point pairs, and *n* is the number of the valid point pairs. The initial values of *D* and *n* are both 0. Then, calculate the average distance of the valid point pairs: $D_{mean}=D/n$. If $D_{mean}<D/n$; thus, the adjustment result of strip *i* and strip *j* is valid. Here, *D*_0_ is a threshold to be set according to the quality of strips. If all strips are valid, it means that the result of strip adjustment is good and can be further evaluated and applied. Otherwise, identify the wrong strips and adjust again.

## 4. Results

The experimental platform is Lenovo ThinkPad E450, made in China. The configuration of the experimental platform is as follows: Intel (R) Xeon (TM) E-2224G 3.50 GHz CPU, 16 GB memory, and Windows 10 operating system. The development platform was Microsoft Visual Studio 2019 C++. This study used the ICP algorithm provided by the PCL (Point Cloud Library) [50], and the version used was PCL 1.8.1. For all of the following experiments, in pair-wise registration, the thresholds $t_{0}$ and $r_{0}$ for the termination of the iteration took the empirical thresholds 0.05 m and 1 × 10^−3^°, respectively. The g2o library [51] was utilized to solve the graph-based optimization.

The experimental data (the relevant information is listed in Table 2) were collected by the Luojia Qilin Cloud II UAV [52], which is independently developed by the Dynamic Mapping Group of the State Key Laboratory of Information Engineering in Surveying, Mapping and Remote Sensing (LIESMARS) of Wuhan University.

### 4.1. Experiment of Strip Adjustment

**Figure 4:**
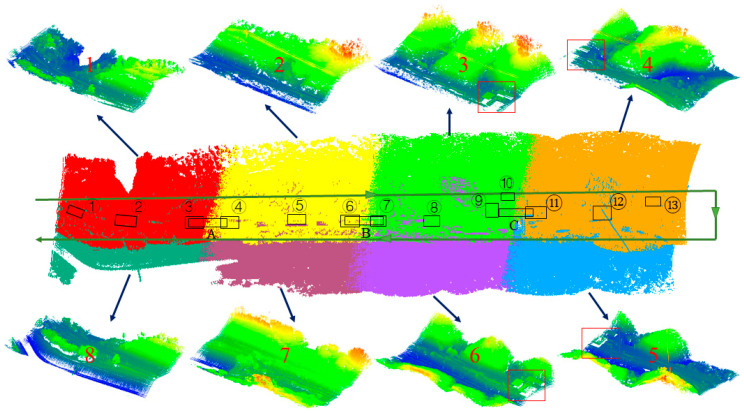
Strip data and serial number after preprocessing. There are continuous hills, undulating terrain, an expressway, an ordinary road, a high-voltage power line, and a toll station (in red rectangles). A more complete experimental region can be seen in Figure 5. The green line is the flight trajectory of the unmanned aerial vehicle (UAV) and the black rectangles mark the position of the planes used to calculate the root-mean-square error (RMSE) in subsequent experiments.

**Figure 5:**
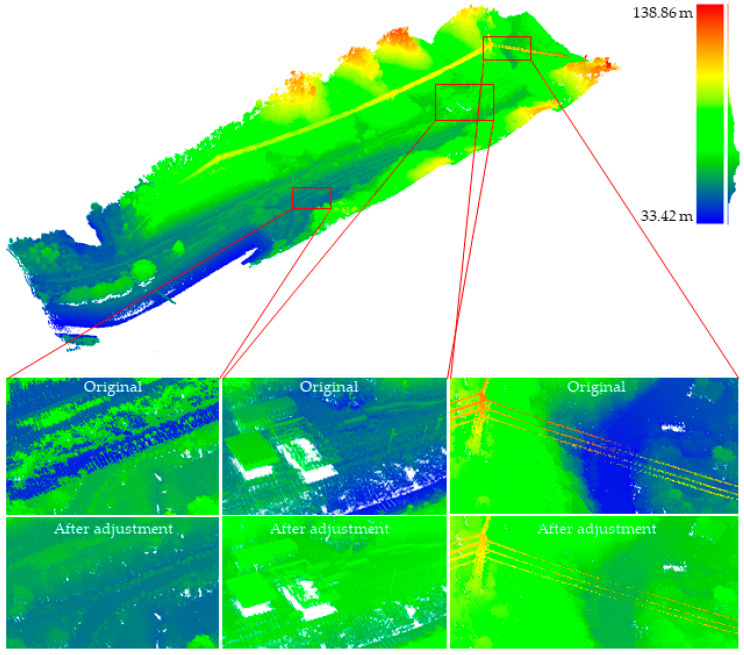
The result after strip adjustment and the comparison of some details.

Based on the workflow described in Section 3, the experiment of strip adjustment was carried out on the experimental data described in Figure 4. In the step of strip registration, the lateral data strip has a large overlap, so the DEM-ICP method was employed for registration, where the length of the DEM grid is 1 × 1 m. The heading strip overlap is low, but it has a good initial alignment. Therefore, the classic ICP algorithm was directly used for the registration of heading strips. The result after strip adjustment and the comparison of some details are shown in Figure 5. From the adjusted data, the road, toll station, high-voltage power line, and other objects can be seen clearly.

#### 4.1.1. Qualitative Evaluation

**Figure 6:**
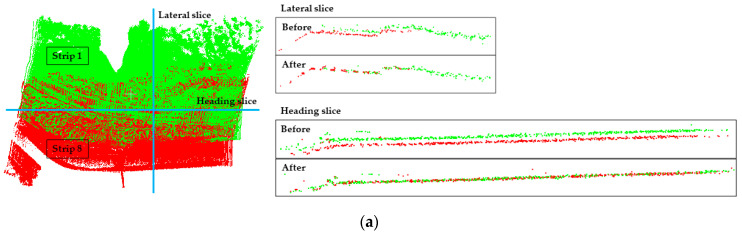

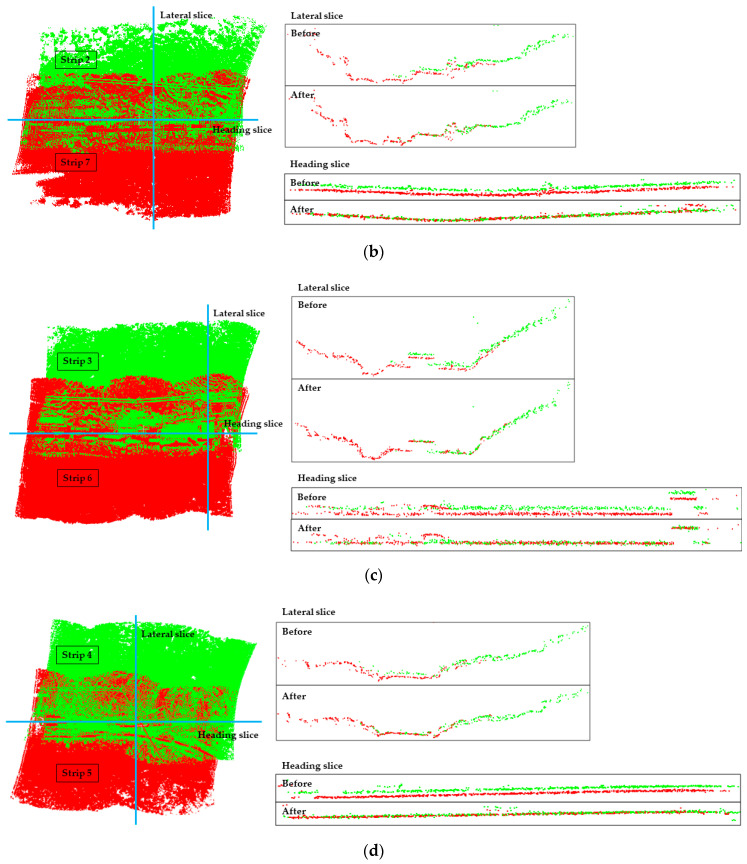
Slice position and results of lateral strip pairs before and after adjustment by the proposed method: (**a**) Strips 1 and 8; (**b**) Strips 2 and 7; (**c**) Strips 3 and 6; (**d**) Strips 4 and 5.

For the purpose of evaluating the results of the strip adjustment experiment, one lateral slice and one heading slice were performed for each pair of registered lateral point clouds (as shown in Figure 6). because one lateral slice and one heading slice can “fix” the registration result well, the accuracy of registration can be qualitatively assessed in all three directions (X, Y, Z). The undulating parts in Figure 6 demonstrate that the strip adjustment method proposed in this paper has good adjustment performance.

#### 4.1.2. Quantitative Evaluation

In addition, to evaluate the adjustment results quantitatively, planes in the overlapping area of the experimental strips were selected for plane fitting, and then the RMSE was calculated. The calculation formula of RMSE is as follows:

(6) $$\mathrm{RMSE}=\sqrt{\frac{1}{n}\overset{n}{\underset{i=1}{\sum }}{\left(x_{i}-x\right)}^{2}}$$

where *x_i_* is the elevation value of the point *i*, *x* is the true value, and *n* is the total number of points in the plane.

**Figure 7:**
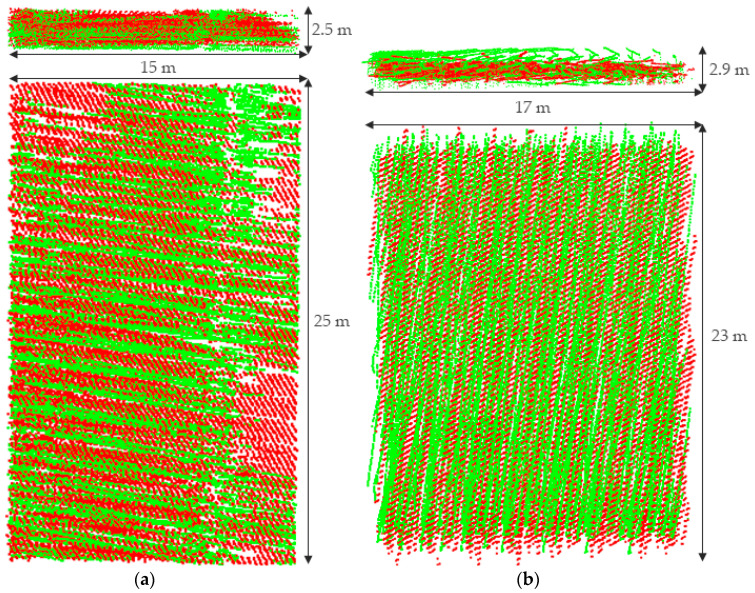
Planes used to calculate the RMSE, where green is Strip 3, and red is Strip 6: (**a**) plane ⑦ with 73,619 points; (**b**) plane ⑨ with 54,758 points.

There were 19 planes used to calculate RMSE, whose distribution is shown in the black rectangles in Figure 4. Because the experimental region is mountainous, most of the selected planes are expressways in the overlapping area between adjacent strips, and only plane ⑨ and plane ⑩ are the roof planes of buildings. Two examples of the selected planes are shown in Figure 7, where Figure 7a is the expressway plane and Figure 7b is the roof plane. Among these, the overlapping area between lateral strips is large, 3–4 planes were selected, and the RMSE was the average value; the overlapping area between heading strips is small, so only a long and narrow plane of the expressway was selected for each pair of registrations to calculate the RMSE, which is marked with letters in Figure 4. It should be noted that plane A, plane B, and plane C were all calculated twice because they fall in the overlapping area of two different heading strips.

**Figure 8:**
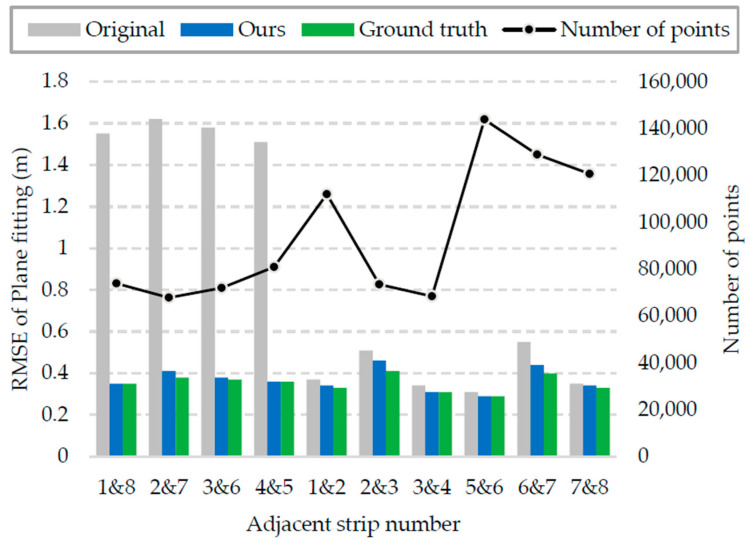
RMSE calculation results, where the grey bar and the blue bar are the RMSE values of each pair of point clouds before and after adjustment, and the green bar is the RMSE value of the ground truth. The black dot is the number of points in the plane where each RMSE value is calculated.

Because the accuracy of the collected strips is related to the accuracy of the acquisition equipment, the range of the experimental region, the scale of the data, and other factors, it is difficult to give a unified standard to evaluate the adjustment results. However, we can register the strips manually, and take the results as the ground truth. The performance of the proposed method can be reflected by comparing its adjustment results with the ground truth. The RMSE calculation results of the original strips, the proposed method, and the ground truth are shown in Figure 8.

As illustrated in Figure 8, compared with the original, the RMSE value between the lateral strips decreased from 1.52–1.62 m to 0.35–0.41 m after adjustment, indicating a 4.2-fold increase in average accuracy; initial alignment between the heading strips was good, but the accuracy was also improved after adjustment. Overall, the average plane fitting RMSE value of the whole experimental strips was less than 0.4 m after adjustment, which is only 0.015 m higher than that of the ground truth, indicating that the proposed method has high accuracy. In the experiment, the initial accuracy of the lateral strip was much lower than that of the heading strip, but after adjustment with our method, the accuracy was equivalent to that of the heading strip. This experimental result demonstrates that the proposed method performs well in adjusting the UAV point clouds in complex mountainous scenes.

### 4.2. Evaluation of the Proposed DEM-ICP Method

In a further effort to evaluate the performance of the proposed DEM-ICP method, Strip 3 and Strip 6 were chosen, which include artificial buildings such as toll stations that facilitate the evaluation of results.

#### 4.2.1. Robustness to Overlap

**Figure 9:**
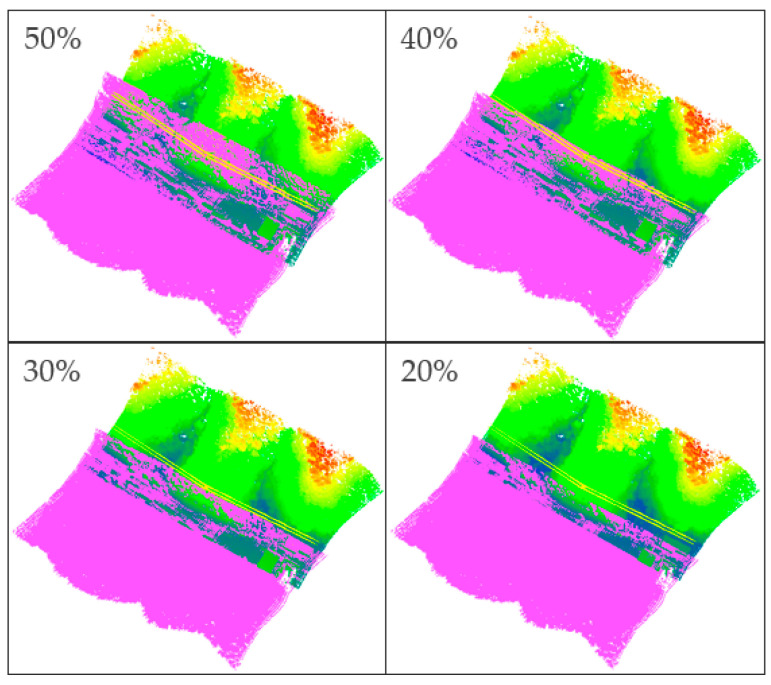
Registration results of different degrees of overlap. The colored point cloud displayed by elevation is Strip 3, and the purple point cloud is Strip 6.

Strip 3 and Strip 6 were cropped so that they overlapped by 50%, 40%, 30%, and 20%, as calculated by Equation (7), where *OA* represents the overlapping area between two adjacent strips, and *TA* represents the total area of both strips. The DEM-ICP method was used for registration, and the influence of the overlap was tested. The registration results are illustrated in Figure 9. For the sake of quantitatively evaluating the registration results, planes ⑦–⑩ as shown in Figure 4 were selected for plane fitting for each pair of registrations, and the average RMSE of four planes is listed in Table 3.

(7) $$overlap=\frac{2*OA}{TA}$$

It can be seen from Table 3 that although the registration accuracy of the DEM-ICP method decreases with the reduction of overlap, the accuracy of the method is not significantly reduced, and it is maintained at a low value, which means that the proposed method has strong robustness to the degree of overlap. The conclusion can be roughly drawn from this experiment that the DEM-ICP method has good performance when there are distinct topographic features and the overlap between neighboring scans is more than 20%.

#### 4.2.2. Performance Comparison

**Figure 10:**
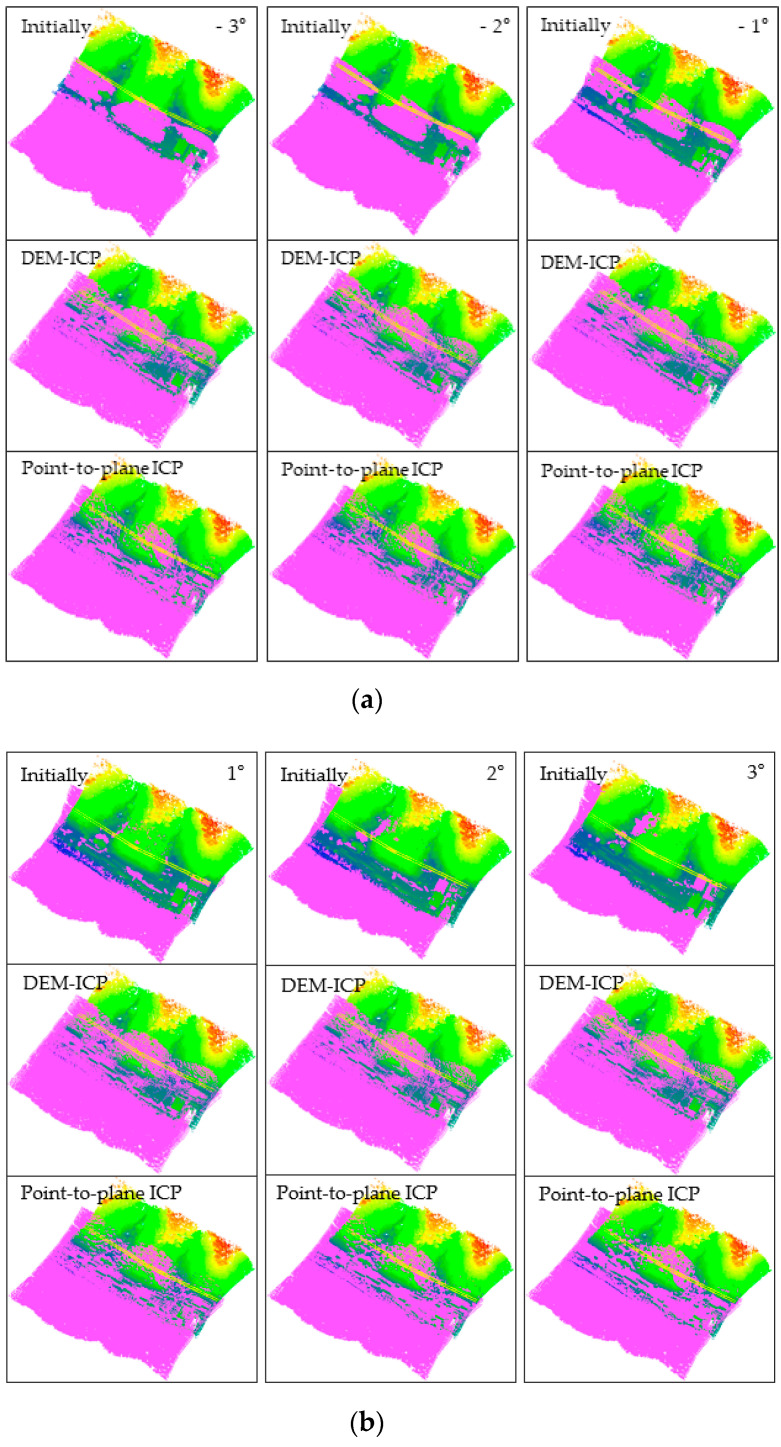
Registration results of the performance comparison experiment. The colored point cloud displayed by elevation is Strip 3, and the purple point cloud is Strip 6: (**a**) the rotation angle θ range from −3° to −1°; (**b**) the rotation angle θ range from 1° to 3°.

Generally, the horizontal accuracy of point clouds collected by a low-cost UAV is relatively low [21]. For this reason, Strip 6 was rotated horizontally around the origin of the coordinate system by Equation (8).

(8) $$R_{Z}\left(\theta \right)=\left[\begin{array}{ccc} \cos\theta & -\sin\theta & 0\\ \sin\theta & \cos\theta & 0\\ 0 & 0 & 1 \end{array}\right]$$

where θ denotes the rotation angle, and $R_{Z}\left(\theta \right)$ is the horizontal rotation matrix. In this experiment, the values of θ were −3°, −2°, −1°, 1°, 2°, and 3°. The transformed results are shown in the purple point clouds marked “Initially” in Figure 10.

**Figure 11:**
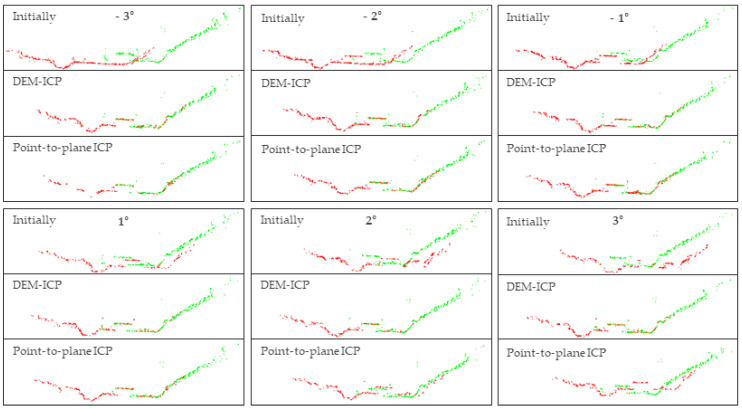
Slices of registration results in the performance comparison experiment. The green point cloud is Strip 3 and the red one is Strip 6. The slice position is the same as the lateral slice in Figure 6c.

The experimental group utilized the DEM-ICP method, and the control group used the method of point-to-plane ICP. Experiment parameters are listed in Table 4 and the experimental results are shown in Figure 10. Eight points of building roof corners were selected from each group of registration results, and the RMSE of the points was calculated. The calculation results are shown in Table 5. In addition, to compare the registration results more intuitively, we also sliced the point clouds before and after registration, as shown in Figure 11.

In this study, only eight points were selected to calculate the RMSE. This was mainly because the experimental area is mountainous, lacking reliable corresponding points. The RMSE was calculated in order to have an index to quantitatively evaluate the registration accuracy in the three directions of XYZ. Furthermore, both Figure 10 and Figure 11 can qualitatively indicate the superiority of the proposed DEM-ICP method.

Analyzing and comparing the registration results of the two methods, we can draw the following conclusions:

1. DEM-ICP is robust, and not sensitive to the accuracy of the initial data. In the above experiments, regardless of the initialization of the given point cloud, the proposed DEM-ICP method can always achieves a satisfactory result. This is because, compared with the unordered point cloud, DEM is well organized, and has higher robustness during pair-wise registration.
  In comparison, it is difficult for the classic point-to-plane ICP method to converge correctly, especially when the two point clouds are close to each other at the beginning (for example, when the rotation angle is 2° and 3° in Figure 10 and Figure 11, the discrepancies of the registration results are obvious). The unsatisfactory results of the point-to-plane ICP method are attributable to the following factors. Firstly, there will be intersections when two point clouds are close to each other, especially in mountainous areas with large vegetation coverage. Then, when the point-to-plane ICP algorithm is applied, these intersecting parts can incorrectly identify the nearest corresponding points, so they may converge to a local minimum or even be non-convergent, and fail to obtain correct results. In addition, it should be noted that these discrepancies cannot be easily eliminated by only increasing the iteration.
2. DEM-ICP has a high time efficiency. The points in Strip 3 and Strip 6 number 4,126,302 and 4,159,645, respectively, whereas the points of Strip 3 and Strip 6 converted into the DEM number only 42,793 and 46,132, respectively. As shown in Table 4, the number of iterations required for the DEM-ICP method is less than 10, which takes less than 1 s in all cases, indicating the rapid convergence speed of DEM-ICP. In contrast, the maximum number of iterations required for point-to-plane ICP is 97 and the time taken is 1299 s; even so, this method still cannot achieve the correct results. In this experiment, the average time cost of a single iteration of the point-to-plane ICP method is about 13.2 s, whereas it is about 0.1 s in the DEM-ICP method; that is, the time incurred in DEM-ICP is only 1/132 times that of point-to-plane ICP.
3. The accuracy of DEM-ICP method is higher. As shown in Table 5, in all cases, the RMSEs of DEM-ICP method in the three directions of XYZ are all less than 0.5 m; these satisfactory results can be seen in Figure 10 and Figure 11. By comparison, when the rotation angle is small (such as −1°), the point-to-plane ICP algorithm has good registration accuracy, but with the increase in rotation angle, the horizontal accuracy of the point-to-plane ICP algorithm is significantly lower than the vertical accuracy. When the rotation angle is ±3°, the maximum RMSE of point-to-plane ICP is as high as 3.32 m, indicating that the registration cannot converge.

## 5. Conclusions

This paper proposes a data-driven strip adjustment method considering point clouds obtained by UAV-borne LiDAR, which mainly includes three steps: data preprocessing, DEM-ICP registration, and graph-based optimization. Experiments show that the proposed method is suitable for strip adjustment in mountainous areas. The main step, DEM-ICP registration, is an improvement of the point-to-plane ICP algorithm. By only using the DEMs generated from ground points, the proposed DEM-ICP has the advantages of good robustness, high registration accuracy, and fast convergence speed, which can be demonstrated from the comparison with point-to-plane ICP method. In addition, this study also verified that DEM-ICP is not sensitive to the overlap between adjacent strips in the case of having obvious topographical features.

However, because the proposed method relies on terrain features, it may not be able to achieve ideal results in flat areas without sufficient topographic features. Scholars have conducted a large number of studies on strip adjustment in urban scenes, but these methods cannot easily handle the data of mountainous areas. Therefore, the method proposed in this paper can be a good supplement for the existing methods.

The workflow of the proposed strip adjustment method is simple and easy to implement, can be used in the strip adjustment of UAV-borne LiDAR data in a large range of mountainous areas, and has practical value in engineering. Because terrain features should be extracted for registration, ground filtering and spatial interpolation are the key steps of the proposed method. Therefore, we believe that our method will perform better if better methods are developed to generate DEMs with higher quality from point clouds. This development deserves further study. In addition, different registration methods [53,54] can also be compared and applied to the proposed method to improve the accuracy and efficiency of pair-wise registration.

## Figures and Tables

**Table 1:** Some of the symbols used in this section and their brief descriptions.

Symbol	Brief Description
*p_i_*, *q_i_*	the *i*th 3D point
*w_i_*	the weight of *p_i_*
*R*	a 3 × 3 rotation matrix
*t*	a 3 × 1 translation vector
*η_i_*	the normal vector of *p_i_*
*T*	a 4 × 4 transformation matrix
*P*	a 4 × 4 pose matrix
*Ω*	the information matrix

**Table 2:** The equipment information used to collect the experimental data, and the relevant information of strip data.

Item	Value	Item	Value
Date	22 April 2019	Area Coverage	980 × 260 m
Place	Guangzhou City, Guangdong Province, China	Point Density	80 pts/m^2^
Laser Scanner	Surestar R-Fans-16	Total Points	28,397,938
IMU	Xsens MTi-300	Number of Strips	8
Flight Height	80 m	Heading Overlap	8%
Flight Velocity	5 m/s	Lateral Overlap	55%

**Table 3:** RMSE calculation results before and after registration.

Overlap	Before Registration (m)	After Registration (m)
50%	1.58	0.425
40%	1.58	0.434
30%	1.58	0.451
20%	1.58	0.454

**Table 4:** Comparison of registration parameters. Here, MSD represents the maximum search distance for corresponding points during registration, which is an important registration parameter. In other words, two different points located on two point clouds; if the distance between them is greater than the MSD, then they will not be recognized as a pair of corresponding points.

Rotation Angle θ	DEM-ICP			Point-to-Plane ICP		
	MSD (m)	Iterations	Time (s)	MSD (m)	Iterations	Time (s)
−3°	10	9	0.801	8	28	368.835
−2°	8	5	0.559	6	24	309.035
−1°	6	2	0.375	5	18	252.738
1°	6	3	0.419	4	20	266.204
2°	8	5	0.546	6	36	445.117
3°	10	9	0.828	8	97	1299.33

**Table 5:** RMSE of selected points in the three directions of XYZ.

Rotation Angle θ	DEM-ICP			Point-to-Plane ICP		
	X (m)	Y (m)	Z (m)	X (m)	Y (m)	Z (m)
−3°	0.49	0.46	0.49	1.54	1.17	0.53
−2°	0.34	0.50	0.35	0.25	0.49	0.54
−1°	0.30	0.47	0.31	0.30	0.33	0.35
1°	0.21	0.44	0.39	0.44	1.46	0.53
2°	0.37	0.46	0.43	1.34	2.60	0.58
3°	0.41	0.48	0.39	1.78	3.32	0.70
